# LETM1 is a potential biomarker of prognosis in lung non-small cell carcinoma

**DOI:** 10.1186/s12885-019-6128-9

**Published:** 2019-09-09

**Authors:** Longzhen Piao, Zhaoting Yang, Ying Feng, Chengye Zhang, Chunai Cui, Yanhua Xuan

**Affiliations:** 1 Department of Oncology, Affiliated Hospital of Yanbian University, No.119 Juzi Road, Yanji, 133002 China; 2 Institute for Regenerative Medicine, Yanbian University College of Medicine, No.977 Gongyuan Road, Yanji, 133002 China; 3 Department of Pathology, Yanbian University College of Medicine, No.977 Gongyuan Road, Yanji, 13302 China; 4 Department of Anatomy, Yanbian University College of Medicine, No.977 Gongyuan Road, Yanji, 13302 China

**Keywords:** Non-small cell lung carcinoma, Leucine zipper-EF-hand-containing transmembrane protein 1, Cancer stemness, Prognosis

## Abstract

**Background:**

Although the leucine zipper-EF-hand-containing transmembrane protein 1 (LETM1) is one of the mitochondrial inner membrane proteins that is involved in cancer prognosis in various tumors, LETM1 as a biomarker for prognostic evaluation of non-small cell lung carcinoma (NSCLC) has not been well studied.

**Methods:**

To address this issue, we used 75 cases NSCLC, 20 cases adjacent normal lung tissues and NSCLC cell lines. We performed immunohistochemistry staining and western blot analysis as well as immunofluorescence imaging.

**Results:**

Our studies show that expression of LETM1 is significantly correlated with the lymph node metastasis (*p* = 0.003) and the clinical stage (*p* = 0.005) of NSCLC. The Kaplan-Meier survival analysis revealed that NSCLC patients with positive expression of LETM1 exhibits a shorter overall survival (OS) rate (p = 0.005). The univariate and multivariate Cox regression analysis indicated that LETM1 is a independent poor prognostic marker of NSCLC. In addition, the LETM1 expression is correlated with cancer stemness-related gene LGR5 (*p* < 0.001) and HIF1α expression (*p* < 0.001), but not with others. Moreover, LETM1 expression was associated with the expression of cyclin D1 (*p* = 0.003), p27 (*p* = 0.001), pPI3K(p85) (*p* = 0.025), and pAkt-Thr308 (*p* = 0.004). Further, our studies show in LETM1-positive NSCLC tissues the microvessel density was significantly higher than in the negative ones (*p* = 0.024).

**Conclusion:**

These results indicate that LETM1 is a potential prognostic biomarker of NSCLC.

**Supplementary information:**

**Supplementary information** accompanies this paper at 10.1186/s12885-019-6128-9.

## Background

Lung cancer is the leading cause of cancer-related deaths worldwide and is one of the most incurable cancers owing to the low rate of curative therapy and high rate of disease relapse [1]. Recent evidence suggests that non-small cell lung carcinoma (NSCLC), like other tumors, harbors cancer stem cell (CSC) populations [2, 3]. NSCLC CSCs, a small subpopulation of cancer cells that possess properties of self-renewal and differentiation into multiple cell types. The presence of cancer stem cells serves as the primary driver for tumor initiation, progression, and metastasis [4–6]. The identification of NSCLC cancer stem cells has been hampered by the lack of robust surface markers. [7]. Thus, define novel marker that represent an effective therapeutic target for NSCLC CSCs is needed. NSCLC cells with CSC characteristics are enriched within populations with specific cell markers such as CD44, CD166, ALDH1A1, Sox2, Oct4, Nanog, and CD133, which also contribute directly to the CSC properties. These markers may be associated with carcinogenesis and tumor progression, and may also play an important role in maintaining the stemness phenotype of CSCs [8–11]. Therefore, studies on CSCs and a better understanding of CSC biology in lung cancer will provide a basis for developing novel diagnostic and therapeutic strategies.

Leucine zipper-EF-hand-containing transmembrane protein 1 (LETM1) is one of the mitochondrial inner membrane proteins that is conserved between yeast and humans [12]. LETM1 acts as an anchor protein and associates with mitochondrial ribosomal protein L36 [13, 14]. In addition, LETM1-mediated inhibition of mitochondrial biogenesis enhances glycolytic ATP supply and activates protein kinase B activity and cell survival signaling [13, 14]. Furthermore, the expression levels of LETM1 markedly increased in various cancers compared with those in normal tissue, demonstrating that high LETM1 expression may be a potential tumor marker [14]. However, the function of LETM1 in tumorigenesis and its regulation are largely unclear, and the role of LETM1 as a prognostic biomarker in NSCLC has not been previously reported. Moreover, some controversies persist regarding the role of LETM1 in lung cancer cells.

In this study, we investigated the clinical significance of LETM1 as a potential NSCLC prognostic marker, LETM1 expression was examined by immunohistochemistry in 75 cases NSCLC and 20 cases adjacent normal lung tissues samples. To evaluate the interaction between LETM1 expression and the stem cell like characteristics of LETM1 positive cells, we analyzed and compared its expression with that of other cancer stemness-related genes such as CD44, LSD1, Sox2 and Sox9. In summary, our studies show that LETM1 expression indicates poor prognosis for NSCLC.

## Methods

### Patients and samples

This study consists of an initial discovery cohort and a clinical validation cohort. In the discovery cohort, we analyzed data from Oncomine database (www.oncomine.org). Bioinformatics analysis were performed using the Oncomine database to analyze mRNA expression. On the other hand, the clinical validation cohort included a total of 95 cases of lung tissue samples including 75 cases of NSCLC and 20 cases of adjacent non-tumor lung tissue (excluded fibrosis, inflammation, dysplasia and interstitial tissues) are obtained from Shanghai Outdo Biotech Co. Ltd. (Outdo Biotech). No patient received preoperative chemotherapy or radiotherapy. Moreover, formalin-fixed and paraffin-embedded sagittal sections of human fetus samples are obtained from Yanbian University Affiliated Hospital. The studies complied with the Helsinki Declaration and were approved by the Human Ethics Committee and the Research Ethics Committee of Yanbian University College of Medicine.

### Immunohistochemical analysis

Tissue sections on microscope slides were deparaffinized, hydrated, and treated with 3% H_2_O_2_ for 15 min to quench endogenous peroxidase activity. Sections were immersed in TE buffer (10 mM Tris and 1 mM EDTA, pH 9.3) for epitope retrieval in a microwave for 30 min. The slides were then incubated with 4% bovine serum albumin for 30 min to block nonspecific immunoreactivity. The sections were then incubated with primary antibodies for 60 min at room temperature. Antibodies used in present study were listed in Additional file 3: Table S1. Sections were then incubated with an anti mouse/rabbit antibody (Envision plus, Dako, Denmark, catalog: K801021–2) for 30 min at room temperature. The chromogen used was ImmPACT AEC Peroxidase Substrate (VECTOR Laboratories) for 20 min. After reading and taking photographs of the slides, sections were then stripped one time used stripping buffer (20% SDS, 0.5 M Tris, and mercaptoethanol) to removing the original antibody for one hour in a water bath at 56 °C to remove the original antibody and then for 10 min in alcohol so that the sections could be restained. Omitting the primary antibody provided negative controls for immunostaining. All the primary antibody stained in the same blots, and in serial sections. All the immunohistochemical staining was evaluated by two pathologists (ZT Yang & YH Xuan) and the staining results were semi-quantitatively scored as negative and positive [15].

The double immunostaining procedure was performed using a two-step method with LETM1 antibody and anti-CD105 antibody (1:250, Abcam, Cambridge, UK, ab170943) to observe the relationship between the expression of LETM1 and microvessel density (MVD) in NSCLC. Primarily, for the LETM1 protocols, except that the chromogen with the 3, 3′-diaminobenzidine (Dako) for 10 min (FLEX20), all steps are the same. Then, subsequent staining of the same section was performed after incubating the samples with an antibody to CD105 by ImmPACT AEC Peroxidase Substrate for 20 min.

### NSCLC cell lines

Three human NSCLC cell lines A549, H1299 and H1650 were purchased from ATCC (Manassas, USA) and maintained in DMEM with high glucose (Life Technologies, Grand Island, NY) containing 10% fetal bovine serum (Life Technologies).

### Chemically induced hypoxia

Hypoxia was achieved by exposing cells cultured in normoxic conditions to cobalt chloride (CoCl_2_) (Sigma-Aldrich, St. Louis, MO, USA). In the present studies, A549 cell line was cultured in DMEM with the CoCl_2_ 100 μmol/l for 6 h, 12 h and 24 h.

### Western blotting

Cells were lysed with RIPA containing with 1 mM PMSF. Then used the BCA protein assay kit was used to measure protein concentrations. The 5 μl marker and 25 μg proteins were separated by 10% SDS-PAGE gels and transferred to PVDF membranes. Membranes were blocked 2 h at RT with 5% skim milk (diluted in TBS), and then incubated with primary antibodies at 4 °C shaking for overnight. Followed by second antibodies anti-rabbit /mouse were blocked 2 h at RT. According to the ECL kit (Enhanced chemiluminescence system kit) protocol, detection was performed.

### Immunofluorescence staining

A549 cells were subcultured in a 6-well plate and incubated at 37 °C 5% CO_2_. After sample preparation by fixation, permeabilization, and blocking, the slides were incubated with primary antibody diluted in 3% BSA at 4 °C overnight. Following primary antibody incubation, the slides were then washed three times and incubated with conjugated secondary antibodies in 3% BSA for 1 h at RT. The slides were washed three times with PBST and counter stained with DAPI (Vector Laboratorise, Burlingame, CA). Immunostained slides were imaged using a confocal laser scanning microscope (Carl Zeiss, Thornwood, New York) and analyzed with Zen software.

### Statistical analysis

A Pearson’s Chi-square (χ2) test was used for significance testing for categorical data. Continuous data are shown as mean ± standard deviation (SD), tested for the differences between groups by one-way analysis of variance (ANOVA). The Kaplan–Meier method and the log-rank test were used for survival analysis. The Cox proportional hazards model was used for multivariate analysis to evaluate the prognostic value of clinicopathologic factors. All tests were two sided, and differences between groups were considered statistically significant at *p*-value of less than 0.05. The SPSS 25.0 statistical software (IBM Singapore Pte Ltd., Registration No.1975–01566-C) was used to conduct the statistical analysis of our data.

## Results

### Expression of LETM1 is correlated with unfavorable progression of NSCLCs

**Fig. 1:**
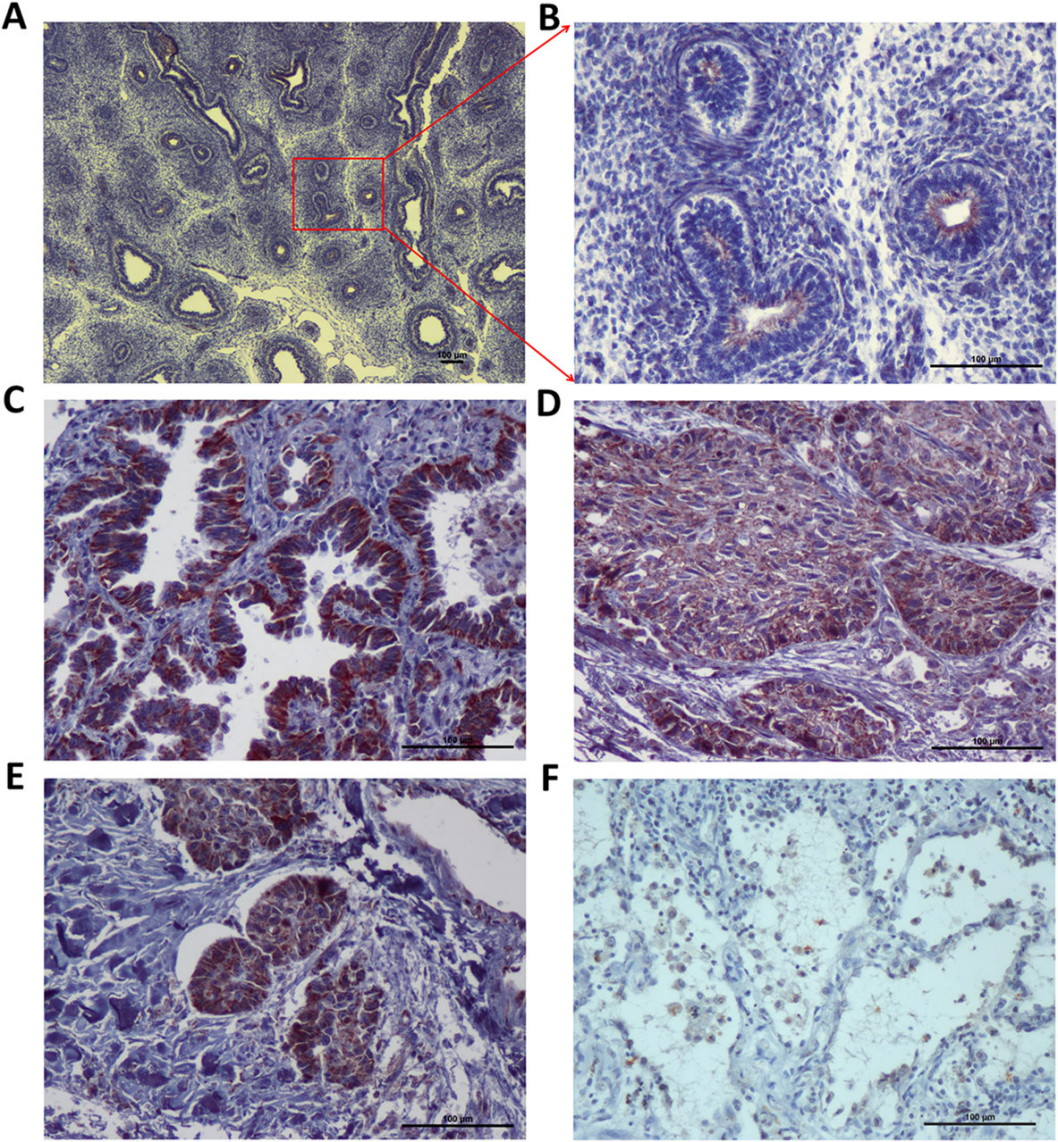
Representative expression of LETM1 in the lung tissues (Immunohistochemical stain). **a** LETM1 expression during lung organogenesis in fetus. **b** Higher magnification of the selected area in a (a, 40×; b, 200×). **c** LETM1 expression in lung adenocarcinoma tissues. **d** LETM1 expression in lung squamous cell carcinoma tissues. **e** LETM1 expression in NSCLC lymphatic invasion area. **f** LETM1 expression in adjacent normal lung tissues (100×)

**Fig. 2:**
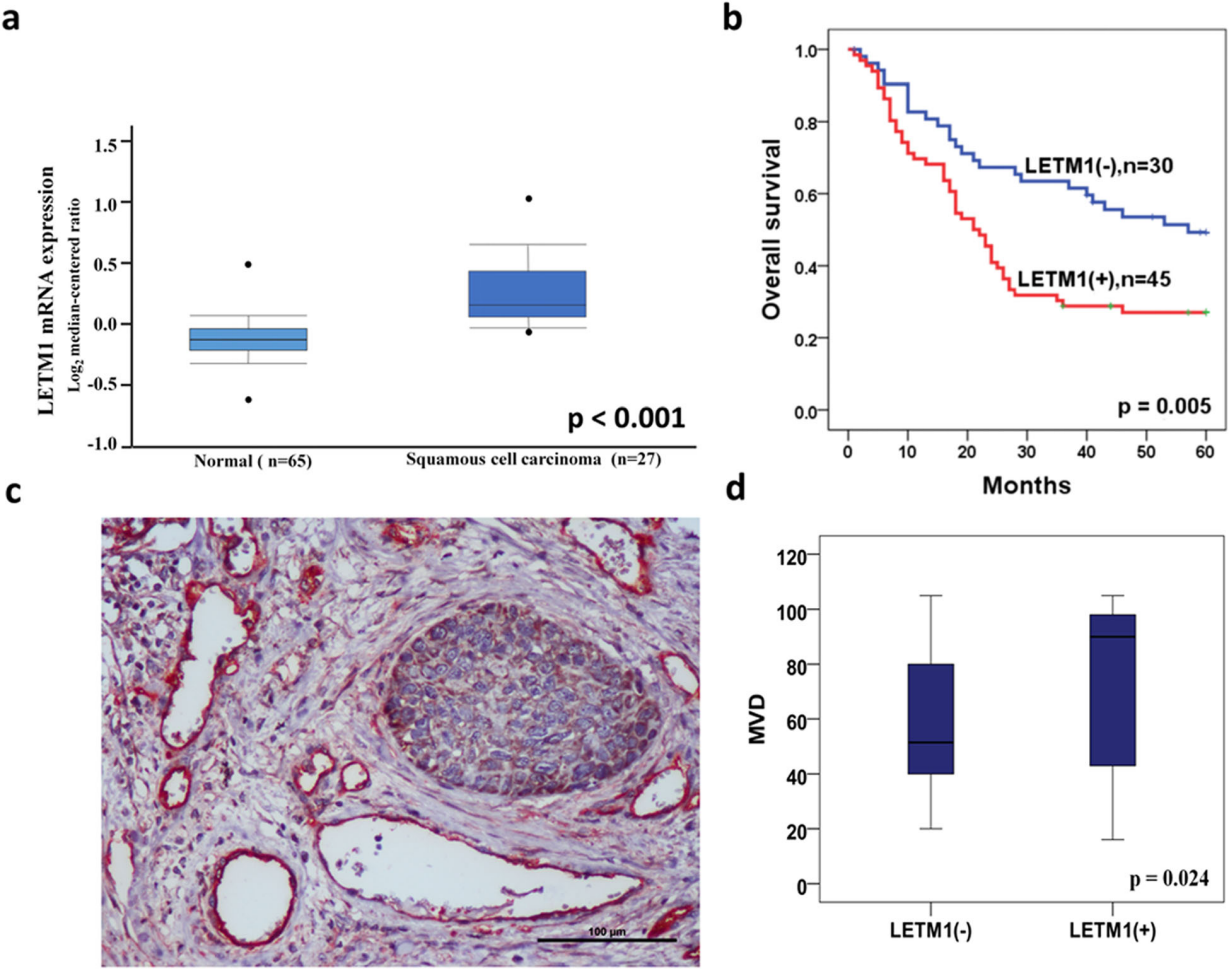
LETM1 expression is correlated with unfavorable progression of non-small cell lung carcinoma (NSCLC). **a** Oncomine mRNA analysis of LETM1 expression in normal and NSCLC (www.oncomine.org) samples. **b** Kaplan-Meier analysis showed overall survival rate of NSCLC patients with LETM1 expression. **c** Immunohistochemical double staining for LETM1/CD105 in NSCLC. LETM1 (brown) is expressed in the cancer cells, and CD105 (red) is expressed in new capillary blood vessels around cancer cells in the host (100×). **d** Graphs showing the microvessel density (MVD) between LETM1 positive and negative groups in NSCLC.

The immunohistochemical study revealed that LETM1 was primarily and abundantly expressed in the lung tissues of fetus (Fig. 1a, b) and in NSCLC tissues (60.0%, 45/75) (Fig. 1c-e), and rarely detectable in adjacent normal lung pulmonary alveoli (0%, 0/20) (Fig. 1f) (*p* < 0.001) (Pearson’s χ2 test). Oncomine mRNA analysis revealed that LETM1 mRNA expression was significantly higher in NSCLC than in normal lung samples (*p* < 0.001) (ANOVA test) (Fig. 2a). LETM1 expression is significantly correlated with the status of lymph node metastasis (*p* = 0.003) and clinical stage (*p* = 0.005) (Table 1) (Pearson’s χ2 test). Our results show that LETM1 expression was diffused and strongly expressed in the lymphatic invasion area of NSCLCs (Fig. 1e). Moreover, the numbers of new capillary blood vessels around the cancer cells significantly higher in cases of LETM1-positive NSCLC compared to that in negative cases (*p* = 0.024) (ANOVA test) (Fig. 2c, d).

**Table 1:** Comparison of clinicopathologic characteristics according to the LETM1 expression in non-small cell lung carcinoma tissues

Variable	n	LETM1 (−) n(%)	LETM1 (+) n(%)	χ^2^	R	*p*-value
Sex				0.186	0.048	0.666
Female	23	8 (34.8)	15 (65.2)			
Male	52	22 (42.3)	30 (57.7)			
Age (years)				1.851	0.149	0.174
≤ 65	35	17 (48.6)	18 (51.4)			
>65	40	13 (32.5)	27 (67.5)			
Size (cm)				0.005	0.008	0.946
≤ 4	43	17 (39.5)	26 (60.5)			
>4	32	13 (40.6)	19 (59.4)			
pT stage				5.774	0.242	0.056
T1	4	2 (50.0)	2 (50.0)			
T2	63	28 (44.4)	35 (55.6)			
T3	8	0 (0.0)	8 (100.0)			
Lymph node metastasis				8.576	0.334	0.003
Negative	56	27 (48.2)	29 (51.8)			
Positive	19	3 (15.8)	16 (84.2)			
Clinical stage				10.772	0.364	0.005
I	55	28 (50.9)	27 (49.1)			
II	16	2 (12.5)	14 (87.5)			
III	4	0 (0.0)	4 (100.0)			

The Kaplan-Meier survival analysis was used to examine whether there is a significant association between LETM1 expression and overall survival (OS) in NSCLC. Our results revealed that LETM1 was a strong prognostic factor in NSCLC. The LETM1 positive group’s median survival time was 28.05 months whereas the negative group’s median survival time was 41.04 months. Specifically, the positive expression of LETM1 in NSCLC patients had significantly lower 5-year OS rates than that in the LETM1 negative groups (*p* = 0.005) (Fig. 2b). Further, the univariate Cox regression analysis show that following factors are significant prognostic factors of poor OS: pT stage (*p* = 0.002), lymph node metastasis (p = 0.002), and LETM1 expression (*p* = 0.006). The multivariate Cox regression analysis show that pT stage (p = 0.005), lymph node metastasis (*p* = 0.012), and LETM1 expression (*p* = 0.008) are adverse independent poor prognostic predictor of NSCLC in terms of OS (Additional file 3: Table S1). These results indicate that LETM1 expression is correlated with the poor progression of NSCLC, and LETM1 is a potential prognostic biomarker of NSCLC.

### LETM1 expression is correlated with the cancer stemness related genes expression in NSCLC

**Fig. 3:**
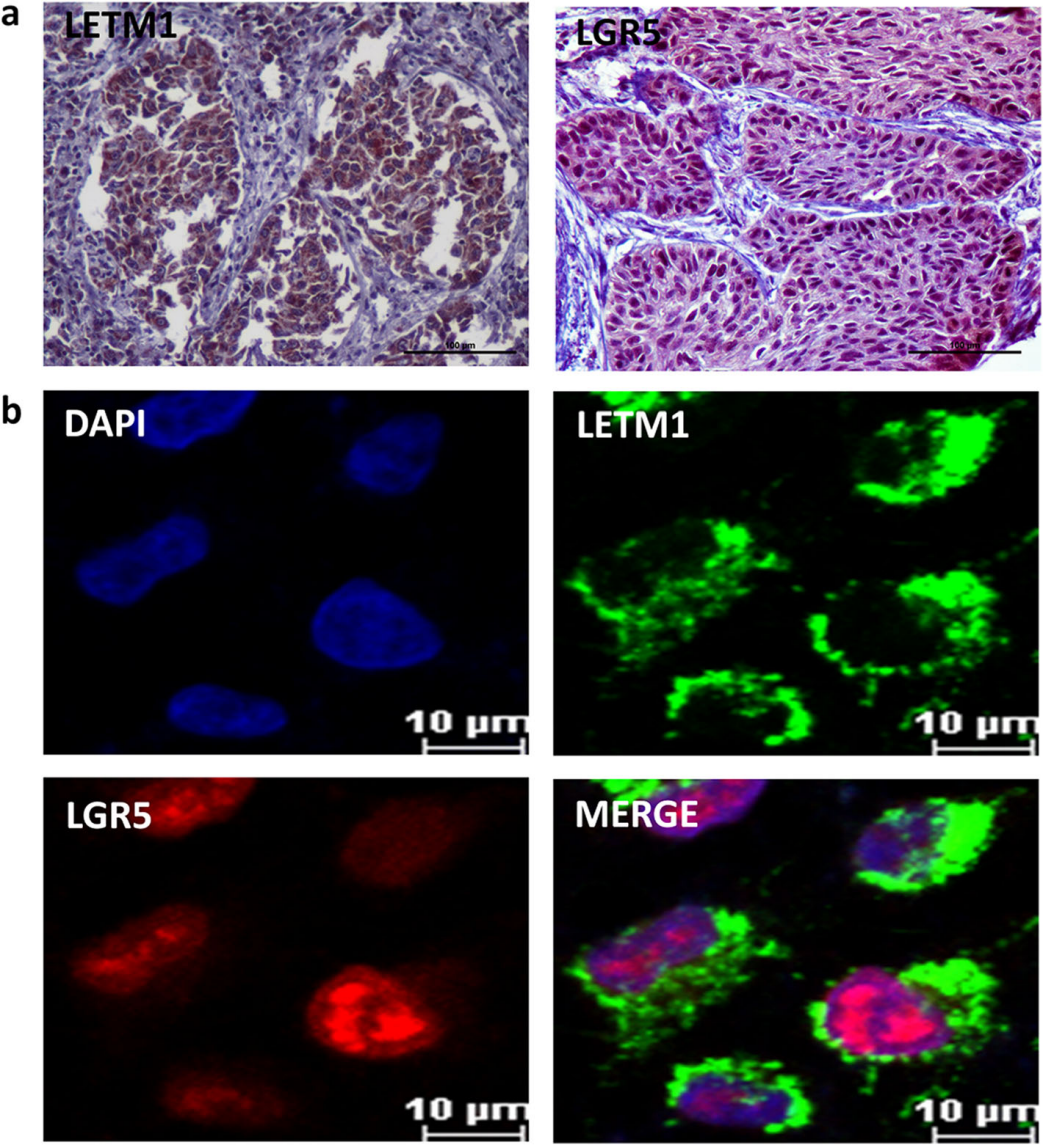
LETM1 and LGR5 expression in non-small cell lung carcinoma (NSCLC) tissues and cancer cells. **a** The expression of LETM1 and LGR5 in NSCLC tissues (Immunohistochemical stain) (100×). **b** Immunofluorescence analysis were performed to detect co-expression of LETM1 and LGR5 in A549 cells. Blue for DAP1; green for LETM1; red for LGR5; double labeling for merged colors

**Fig. 4:**
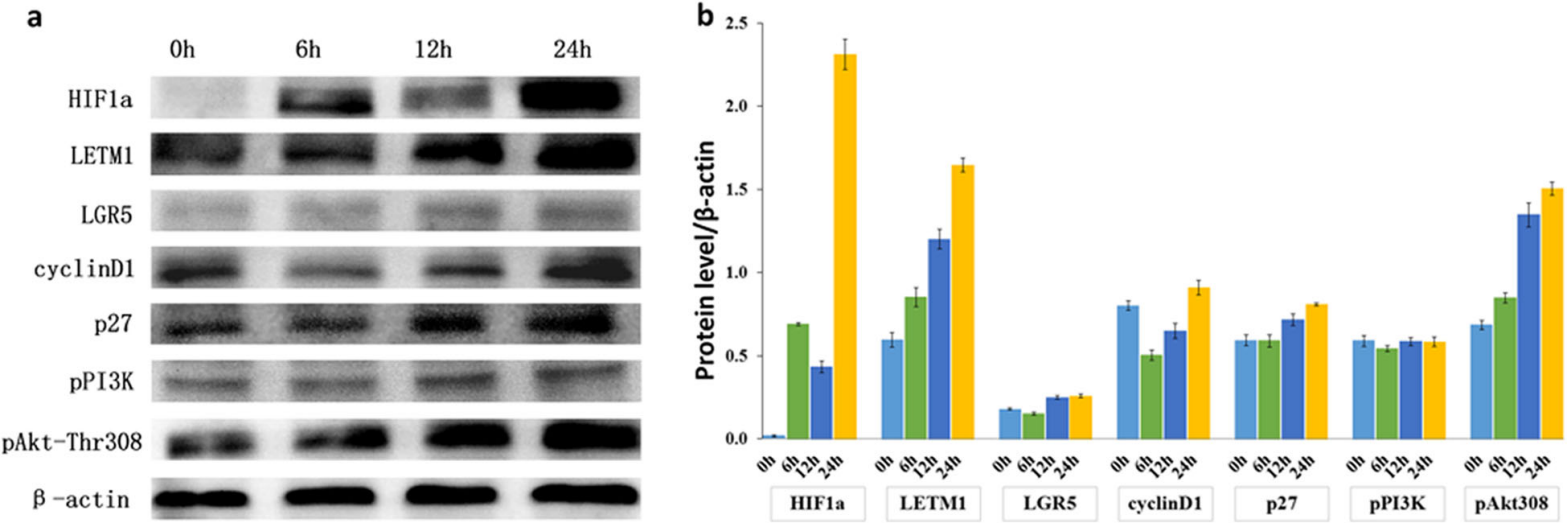
LETM1 expression is correlated with cell cycle and PI3K/Akt signaling related genes expression in non-small cell lung carcinoma (NSCLC) tissues. **a** Western blot analysis of the protein levels of HIF1α, LETM1, LGR5, cyclin D1, p27, pPI3K (p85), and pAkt-Thr308 in A549 cells under hypoxia conditions. β-actin was used as a loading control. **b** Blot signals were quantified using ImageJ program. Results were normalized by β-actin signals

In order to determine if LETM1 expression is associated with the cancer stemness in NSCLC, we investigated the correlation between LETM1 and cancer stemness related genes expression in NSCLC. Our studies show that stemness related genes, such as CD44, CD133, LGR5, LSD1, OCT4, Sox2 and Sox9 were co-upregulated with LETM1 in A549 cells compared to H1299 and H1650 cells (Additional file 2: Figure S2a, b) (ANOVA test). To further verify the above observations, we examined the expression of LETM1 and stemness related genes in NSCLC tissues. The immunohistochemical study revealed that LETM1 expression is associated with the expression of stemness-related gene LGR5 and HIF1α (both p < 0.001), but not with others (Table 2, Additional file 1: Figure S1) (Pearson’s χ2 test). LETM1 is mainly expressed in the cytoplasm and LGR5 is mainly expressed in the nucleus of NSCLC cells (Fig. 3a, b). Further, the LETM1 is co-expressed with the LGR5 in A549 cell line, as revealed by immunofluorescence (Fig. 3b). Hypoxic microenvironment plays important roles in maintenance of cancer stem cells [16]. Therefore, we tested whether hypoxic condition would promote LETM1 and cancer stemness gene LGR5 expression in NSCLC cells. When A549 cells were exposed to CoCl_2_ for 6 h, 12 h, and 24 h, the protein expression levels of HIF1α, LETM1 and LGR5 were higher than those in cells under normoxia (*p* < 0.001 and *p* < 0.001, respectively) (Fig. 4a, b) (ANOVA test). Taken together, these results indicate that LETM1 may be an important factor associated with cancer stemness.

**Table 2:** Correlation of LETM1 expression with cancer stem cell makers expression in non-small cell lung carcinoma tissues

Variable	n	LETM1 (−) n(%)	LETM1 (+) n(%)	χ^2^	R	*p*-value
Sox2				0.436	−0.076	0.509
Negative	36	13 (36.1)	23 (63.9)			
Positive	39	17 (43.6)	22 (56.4)			
Sox9				1.862	0.158	0.172
Negative	28	14 (50.0)	14 (50.0)			
Positive	47	16 (34.0)	31 (66.0)			
LSD1				0.009	0.011	0.924
Negative	42	17 (40.5)	25 (59.5)			
Positive	33	13 (39.4)	20 (60.6)			
CD44				0.067	−0.031	0.796
Negative	35	13 (37.1)	22 (62.9)			
Positive	40	17 (42.5)	23 (57.5)			
CD133				0.195	0.050	0.659
Negative	16	7 (43.8)	9 (56.3)			
Positive	59	23 (39.0)	36 (61.0)			
LGR5				13.187	0.417	<0.001
Negative	25	16 (64.0)	9 (36.0)			
Positive	50	14 (28.0)	36 (72.0)			
HIF-1α				13.062	0.423	<0.001
Negative	25	18 (72.0)	7 (28.0)			
Positive	50	12 (24.0)	38 (76.0)

### LETM1 expression is associated with cell cycle regulatory genes and PI3K/Akt signaling gene expression in NSCLC

Cell cycle progression and PI3K/Akt signaling is a key regulator of cell survival during tumor promotion. Our immunohistochemical staining revealed that LETM1 expression is positively associated with the cell cycle regulatory genes and PI3K/Akt signaling genes, such as cyclin D1 (*p* = 0.003), p27 (*p* = 0.001), pPI3K (p85) (*p* = 0.025), and pAkt-Thr308 (*p* = 0.004) expression in NSCLC tissues (Additional file 3: Table S2) (Pearson’s χ2 test). When A549 cells were exposed to CoCl_2_ for 6 h and 12 h, the protein expression levels of p27 and pAkt-Thr308 were higher than those in cells under normoxia (p = 0.003 and *p* < 0.001, respectively) (Fig. 4a,b) (ANOVA test). These results indicate that expression of LETM1 is positively associated with the expression of cell cycle related genes and activation of PI3K/Akt signaling in NSCLC cells.

## Discussion

In this study, we describe the expression of LETM1 in lung cancer cells as a reliable marker of poor prognosis for patients with NSCLC. Our studies show that a positive association between the expression of LETM1 with LGR5 and HIF1α in NSCLC. In addition, the simultaneous expression of LETM1 is associated with cyclin D1, p27, pPI3K (p85), and pAkt-Thr308. Thus, our results indicate that LETM1 plays an important role in the progression of NSCLC.

Immunohistochemical studies revealed that LETM1 was abundantly expressed in NSCLC tissues, and rarely expressed in adjacent non-tumor lung pulmonary alveoli, indicating that LETM1 potentially plays an important role in NSCLC development (Fig. 1). In triple-negative breast cancer, the LETM1 expression is significantly associated with histological grade, clinical stage, and lymph node metastasis [17]. However, Hwang et al. reported that overexpression of LETM1 could induce mitochondrial destruction of lung cancer cells and facilitate apoptosis, suggesting that LETM1 upregulation may play a key role in suppressing lung cancer growth and progression [18]. On the contrary, our results revealed that LETM1 expression is significantly associated with lymph node metastasis and advanced clinical stage (Table 1). Moreover, LETM1 expression was diffuse and strongly expressed in the lymphatic invasion area of NSCLC (Fig. 1). These results suggest that LETM1 maybe promotes the invasion or metastasis of NSCLC cells. Notably, angiogenesis is a key tumorigenic phenomenon for cancer progression. Our studies show that the MVD was significantly higher in NSCLCs positive for LETM1 expression, suggesting that LETM1 expression is correlated with the angiogenesis of NSCLC (Fig. 2). The phenomenon suggests that dysregulation of LETM1 has far-reaching influence in the dysfunction of lung cancer cells. Here, we also found that LETM1 is strongly associated with shortened OS rate of patients with NSCLC (Fig. 2). A similar trend was reported in triple-negative breast cancer [17]. Overall, our results suggest that the upregulation of LETM1 expression in NSCLC may play a key role in tumor growth and cancer cell proliferation, leading to poor prognosis.

LGR5 has 18 leucine-rich repeats and 7 transmembrane regions, and is a member of the G protein-coupled receptor superfamily. Furthermore, LGR5 has been reported to be a CSC surface marker of colorectal carcinogenesis and a target gene of the Wnt signaling pathway [19]. Previous studies have suggested that LGR5 expression is an independent prognostic marker in NSCLC [20]. Since ALDH1A1 was aberrantly expressed in LGR5-positive NSCLC cells, LGR5 may be a novel marker of NSCLC stem-like cells [20]. Further, hypoxic conditions play important roles in maintenance of CSC features [16]. Our studies show that LETM1 expression is positively associated with HIF1α as well as LGR5 expression in NSCLC tissues (Table 2). In hypoxic conditions expression levels of HIF1α, LETM1 and LGR5 were higher than those in cells under normoxia (Fig. 4). Immunofluorescence showed that LETM1 significantly co-stained with LGR5 in A549 cells (Fig. 3). Moreover, cancer stemness related genes such as CD44, CD133, LGR5, LSD1, OCT4, Sox2 and Sox9 were co-upregulated with LETM1 in A549 cells (Additional file 2: Figure S2). These results indicate that LETM1 is a potential cancer stemness associated gene in NSCLC. However, further studies are required to elucidate the link between LETM1 expression and CSCs in NSCLC.

It was reported that silencing of LETM1 expression affects autophagy activity and induces AMPK activation and cell cycle arrest [21]. Furthermore, LETM1 enhances PKB/Akt activation by inhibition of C-terminal modulator protein (CTMP). LETM1 and CTMP participate in insulin signaling via regulation of PKB/Akt activity [22]. LETM1 is associated with mitochondrial function and PKB/Akt signaling, and LETM1 overexpression increased Akt and pAkt in human papillary thyroid carcinoma [23]. Our results show that LETM1 positively correlated with cyclin D1, p27, pPI3K (p85), and pAkt-Thr308 expression in NSCLC (Additional file 3: Table S2). These results indicate that LETM1 may have a crucial role in NSCLC cell cycle progression through regulation of cell cycle related proteins and PI3K/Akt signaling pathway.

## Conclusion

Taken together, our studies strongly indicate that the expression of LETM1 is positively associated with cancer stemness-related gene expression in NSCLC.

## Supplementary information

**Additional file 1: Figure S1**. Immunohistochemical staining of cancer stemness related genes in non-small cell lung carcinoma tissues. (a) LGR5, (b) CD133, (c) CD44, (d) LSD1, (e) Sox2, and (f) Sox9 (100×). (TIF 3020 kb)

**Additional file 2: Figure S2**. ETM1 and cancer stemness related genes expressed in non-small cell lung carcinoma cells. (a) Western blot analysis to determine protein levels of LETM1 and cancer stemness related genes expressed in A549, H1299 and H1650 cells. β-actin was used as a loading control. (b) Blot signals were quantified using ImageJ program. Results were normalized by β-actin signals. (TIFF 195 kb)

**Additional file 3: Table S1.** Antibodies in this study. **Table S2.** Univariate and Multivariate analyses for prognostic variables of overall survival in non-small cell lung carcinoma patients using Cox proportional-hazards regression. **Table S3.** Correlation of LETM1 expression with cell cycle genes expression in non-small cell lung carcinoma tissues. (DOCX 24 kb)

## Data Availability

The datasets used and/or analysed during the current study are available from the corresponding author on reasonable request.

## Abbreviations

CI: Confidence interval
CSC: Cancer stem cell
HR: Hazard ratio
LETM1: Leucine zipper-EF-hand-containing transmembrane protein 1
NSCLC: Non-small cell lung carcinoma
OS: Overall survival
pT: Primary tumor

## References

[1] Torre LA, Bray F, Siegel RL, Ferlay J, Lortet-Tieulent J, Jemal A (2015). Global cancer statistics 2012. CA Cancer J Clin.

[2] Ye Ting, Li Jingyuan, Sun Zhiwei, Liu Yongli, Kong Liangsheng, Zhou Shixia, Tang Junlin, Wang Jianyu, Xing H. Rosie (2019). Nr5a2 promotes cancer stem cell properties and tumorigenesis in nonsmall cell lung cancer by regulating Nanog. Cancer Medicine.

[3] Sui X, Geng JH, Li YH, Zhu GY, Wang WH (2018). Calcium channel α2δ1 subunit (CACNA2D1) enhances radioresistance in cancer stem-like cells in non-small cell lung cancer cell lines. Cancer Manag Res.

[4] Lee JT, Herlyn M (2007). Old disease, new culprit: tumor stem cells in cancer. J Cell Physiol.

[5] Frank NY, Schatton T, Frank MH (2010). The therapeutic promise of the cancer stem cell concept. J Clin Invest.

[6] Todaro M, Francipane MG, Medema JP, Stassi G (2010). Colon cancer stem cells: promise of targeted therapy. Gastroenterology..

[7] Giangreco A, Groot KR, Janes SM (2007). Lung cancer and lung stem cells: strange bedfellows?. Am J Respir Crit Care Med.

[8] Navas Tony, Pfister Thomas D., Colantonio Simona, Aziz Amina, Dieckman Lynda, Saul Richard G., Kaczmarczyk Jan, Borgel Suzanne, Alcoser Sergio Y., Hollingshead Melinda G., Lee Young H., Bottaro Donald P., Hiltke Tara, Whiteley Gordon, Takebe Naoko, Kinders Robert J., Parchment Ralph E., Tomaszewski Joseph E., Doroshow James H. (2018). Novel antibody reagents for characterization of drug- and tumor microenvironment-induced changes in epithelial-mesenchymal transition and cancer stem cells. PLOS ONE.

[9] Du W, Ni L, Liu B, Wei Y, Lv Y, Qiang S, Dong J, Liu X. Upregulation of SALL4 by EGFR activation regulates the stemness of CD44-positive lung cancer. Oncogenesis. 2018;7(4). 10.1038/s41389-018-0045-7. 10.1038/s41389-018-0045-7 PMC5915399 29691367

[10] Chen W, An J, Guo J, Wu Y, Yang L, Dai J, Gong K, Miao S, Xi S, Du J (2018). Sodium selenite attenuates lung adenocarcinoma progression by repressing SOX2-mediated stemness. Cancer Chemother Pharmacol.

[11] Phiboonchaiyanan PP, Chanvorachote P (2017). Suppression of a cancer stem-like phenotype mediated by alpha-lipoic acid in human lung cancer cells through down-regulation of β-catenin and Oct-4. Cell Oncol (Dordr).

[12] Schlickum S, Moghekar A, Simpson JC, Steglich C, O'Brien RJ, Winterpacht A, Endele SU (2004). LETM1, a gene deleted in wolf-Hirschhorn syndrome, encodes an evolutionarily conserved mitochondrial protein. Genomics..

[13] Frazier AE, Taylor RD, Mick DU, Warscheid B, Stoepel N, Meyer HE, Ryan MT, Guiard B, Rehling P (2006). Mdm38 interacts with ribosomes and is a component of the mitochondrial protein export machinery. J Cell Biol.

[14] Piao L, Li Y, Kim SJ, Byun HS, Huang SM, Hwang SK, Yang KJ, Park KA, Won M, Hong J, Hur GM, Seok JH, Shong M, Cho MH, Brazil DP, Hemmings BA, Park J (2009). Association of LETM1 andmrpl36 contributes to the regulation of mitochondrial ATP production andnecrotic cell death. Cancer Res.

[15] Yang Zhao-Ting, Yeo So-Young, Yin Yong-Xue, Lin Zhen-Hua, Lee Hak-Min, Xuan Yan-Hua, Cui Yan, Kim Seok-Hyung (2016). Tenascin-C, a Prognostic Determinant of Esophageal Squamous Cell Carcinoma. PLOS ONE.

[16] Schöning JP, Monteiro M, Gu W (2017). Drug resistance and cancer stem cells the shared but distinct roles of hypoxia-inducible factors HIF1α and HIF2α. Clin Exp Pharmacol Physiol.

[17] Wang CA, Liu Q, Chen Y, Liu S, Xu J, Cui X, Zhang Y, Piao L (2015). Clinical implication of leucine zipper/EF hand-containing transmembrane-1 overexpression in the prognosis of triple-negative breast cancer. Exp Mol Pathol.

[18] Hwang SK, Piao L, Lim HT, Minai-Tehrani A, Yu KN, Ha YC, Chae CH, Lee KH, Beck GR, Park J, Cho MH (2010). Suppression of lung tumorigenesis by leucine zipper/EF hand-containing transmembrane-1. PLoS One.

[19] Barker N, van Es JH, Kuipers J, Kujala P, van den Born M, Cozijnsen M, Haegebarth A, Korving J, Begthel H, Peters PJ, Clevers H (2007). Identification of stem cells in small intestine and colon by marker gene LGR5. Nature..

[20] Gao F, Zhou B, Xu JC, Gao X, Li SX, Zhu GC, Zhang XG, Yang C (2015). The role of LGR5 and ALDH1A1 in non-small cell lung cancer: Cancer progression and prognosis. Biochem Biophys Res Commun.

[21] Doonan PJ, Chandramoorthy HC, Hoffman NE, Zhang X, Cardenas C, Shanmughapriya S, Rajan S, Vallem S, Chen X, Foskett JK, Cheung JY, Houser SR, Madesh M (2014). LETM1-dependent mitochondrial Ca2+ flux modulates cellular bioenergetics and proliferation. FASEB J.

[22] Park J, Li Y, Kim SH, Yang KJ, Kong G, Shrestha R, Tran Q, Park KA, Jeon J, Hur GM, Lee CH, Kim DH, Park J (2014). New players in high fat diet-induced obesity: LETM1 and CTMP. Metabolism..

[23] Lee J, Lee WK, Seol MY, Lee SG, Kim D, Kim H, Park J, Jung SG, Chung WY, Lee EJ, Jo YS (2016). Coupling of LETM1 up-regulation with oxidative phosphorylation and platelet-derived growth factor receptor signaling via YAP1 transactivation. Oncotarget..

