# Supplementary figures and images for: LETM1 is a potential biomarker of prognosis in lung non-small cell carcinoma
Source: BMC Cancer. 2019 Sep 9;19:898. doi: 10.1186/s12885-019-6128-9 (PMC6734262; doi:10.1186/s12885-019-6128-9)

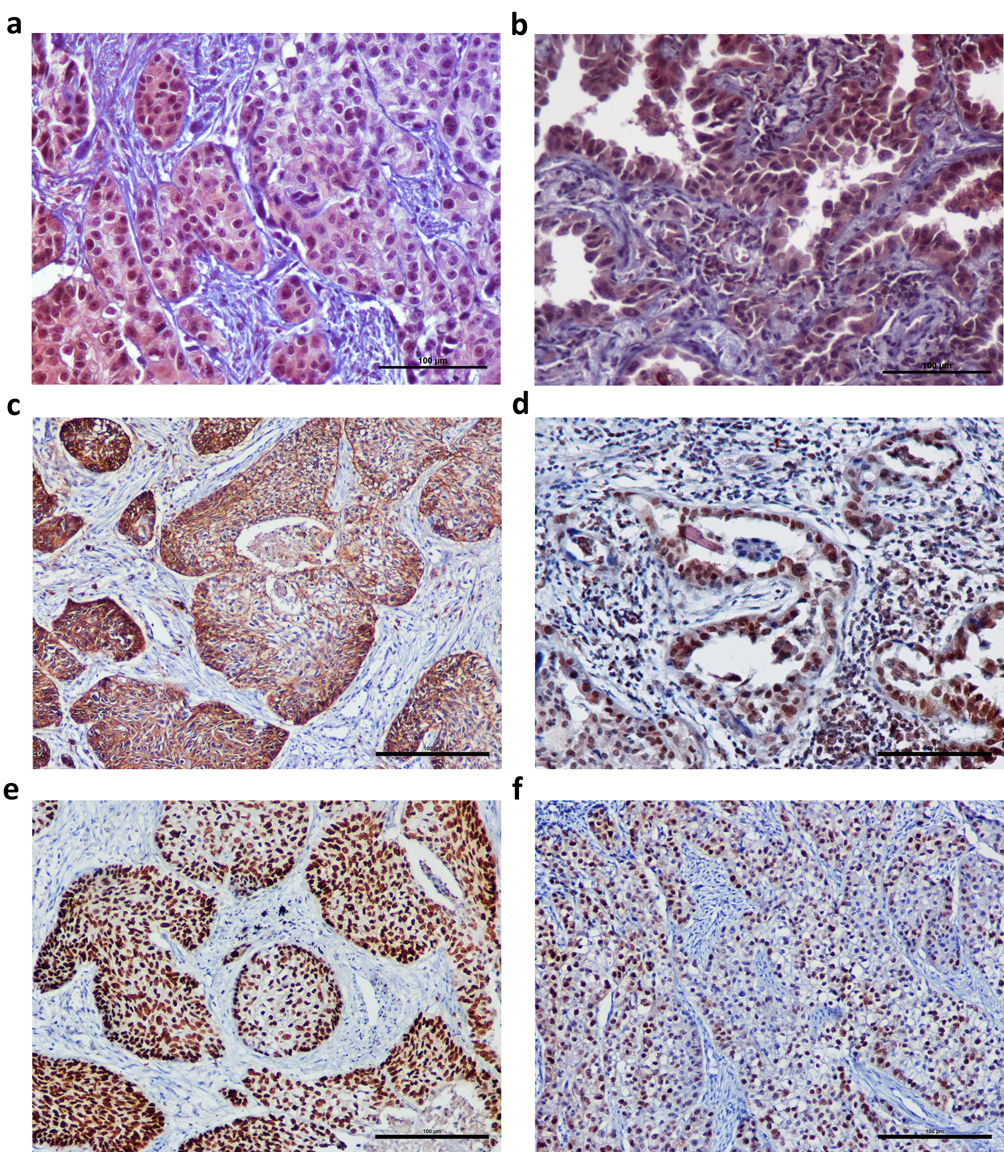

Supplement: Supplementary file 1 — Additional file 1: Figure S1. Immunohistochemical staining of cancer stemness related genes in non-small cell lung carcinoma tissues. (a) LGR5, (b) CD133, (c) CD44, (d) LSD1, (e) Sox2, and (f) Sox9 (100×). (TIF 3020 kb) [file 12885_2019_6128_MOESM1_ESM.tif]

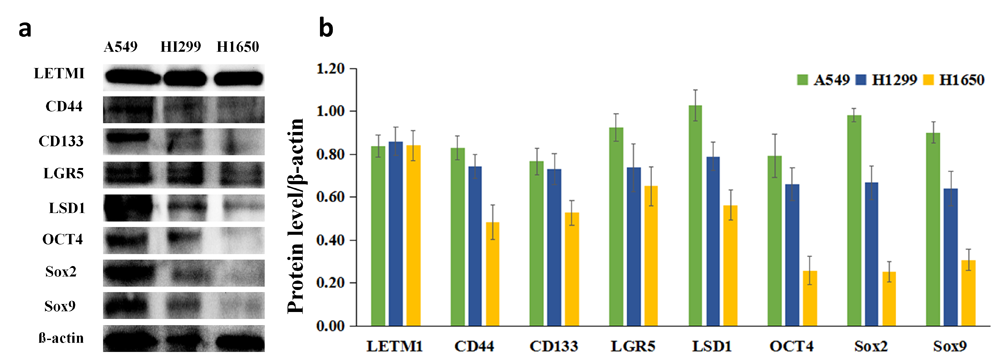

Supplement: Supplementary file 2 — Additional file 2: Figure S2. ETM1 and cancer stemness related genes expressed in non-small cell lung carcinoma cells. (a) Western blot analysis to determine protein levels of LETM1 and cancer stemness related genes expressed in A549, H1299 and H1650 cells. β-actin was used as a loading control. (b) Blot signals were quantified using ImageJ program. Results were normalized by β-actin signals. (TIFF 195 kb) [file 12885_2019_6128_MOESM2_ESM.tiff]
