# Supplementary material for: LETM1 is a potential biomarker of prognosis in lung non-small cell carcinoma
Source: BMC Cancer. 2019 Sep 9;19:898. doi: 10.1186/s12885-019-6128-9 (PMC6734262; doi:10.1186/s12885-019-6128-9)
Supplement: Supplementary file 3 — Additional file 3: Table S1. Antibodies in this study. Table S2. Univariate and Multivariate analyses for prognostic variables of overall survival in non-small cell lung carcinoma patients using Cox proportional-hazards regression. Table S3. Correlation of LETM1 expression with cell cycle genes expression in non-small cell lung carcinoma tissues. (DOCX 24 kb) [file 12885_2019_6128_MOESM3_ESM.docx]

**Table S1** Antibodies in this study.

| Antibodies against | Company | Catalog number |
| --- | --- | --- |
| LETM1 | Abnova, Taiwan | H00003954-M03 |
| Sox2 | R&D, USA | AF2018 |
| Sox9 | Abnova, USA | H00006662-M04 |
| CD44 | Abcam, UK | ab51037 |
| LSD1 | Sigma, USA | L7293 |
| LGR5 | Abcam, UK | ab199335 |
| HIF1α | Millipore, USA | MAB5382 |
| p21 | Abcam, UK | ab109520 |
| p27 | Millipore, USA | 06-445 |
| cyclinD1 | Abcam, UK | ab16663 |
| pPI3K(p85) | Abcam, UK | ab86714 |
| pAkt-Ser473 | Millipore, USA | 17-457 |
| pAkt-Thr308, | Millipore, USA | 46-645MAG |
| NF-κB p65 | Millipore, USA | 04-1008 |
| LC3A | Cell Signaling Technology, USA | 12741 |
| β-actin | Bioss, China | bs-0061R |

**Table S2** Univariate and Multivariate analyses for prognostic variables of overall survival in non-small cell lung carcinoma patients using Cox proportional-hazards regression.

| Characteristic | Univariate | | |  | Multivariate | | |
| --- | --- | --- | --- | --- | --- | --- | --- |
|  | HR | 95% CI | p-value |  | HR | 95% CI | p-value |
| Age(years) |  |  | 0.079 |  |  |  | 0.364 |
| ﹤65 | 1.00 |  | - |  | 1.00 |  | - |
| ≥65 | 1.709 | 0.941-3.105 |  |  | 1.360 | 0.700-2.640 |  |
| Size（cm） |  |  | 0.774 |  |  |  | 0.570 |
| ﹤4 | 1.00 |  | - |  | 1.00 |  | - |
| ≥4 | 1.132 | 0.722-1.774 |  |  | 0.870 | 0.539-1.405 |  |
| pT stage |  |  | 0.002 |  |  |  | 0.002 |
| T1 | 1.00 |  | - |  | 1.00 |  | - |
| T2 | 5.018 | 1.099-22.912 |  |  | 3.678 | 0.764-17.694 |  |
| T3 | 9.556 | 2.337-39.070 |  |  | 7.570 | 1.787-32.070 |  |
| Lymph node metastasis |  |  | 0.002 |  |  |  | 0.012 |
| Negative | 1.00 |  | - |  | 1.00 |  | - |
| Positive | 2.317 | 1.378-3.893 |  |  | 2.003 | 1.164-3.445 |  |
| LTEM1 |  |  | 0.006 |  |  |  | 0.008 |
| Negative | 1.00 |  | - |  | 1.00 |  | - |
| Positive | 1.966 | 1.216-3.181 |  |  | 1.934 | 1.185-3.155 |  |

**Table S3** Correlation of LETM1 expression with cell cycle genes expression in non-small cell lung carcinoma tissues.

| Variable | n | LETM1 (-) n(%) | LETM1 (+) n(%) | χ^2^ | p-value |
| --- | --- | --- | --- | --- | --- |
| Survivin |  |  |  | 0.000 | 0.994 |
| Negative | 27 | 11(42.3) | 16(59.3) |  |  |
| Positive | 48 | 19(39.6) | 29(60.4) |  |  |
| Cyclin D1 |  |  |  | 8.790 | 0.003 |
| Negative | 23 | 15(65.2) | 8(34.8) |  |  |
| Positive | 52 | 15(28.8) | 37(71.2) |  |  |
| p16 |  |  |  | 0.769 | 0.380 |
| Negative | 28 | 13(46.4) | 15(53.6) |  |  |
| Positive | 47 | 17(36.2) | 30(63.8) |  |  |
| p21 |  |  |  | 0.000 | 1.000 |
| Negative | 25 | 10(40.0) | 15(60.0) |  |  |
| Positive | 50 | 20(40.0) | 30(60.0) |  |  |
| p27 |  |  |  | 10.685 | 0.001 |
| Negative | 26 | 17(65.4) | 9(34.6) |  |  |
| Positive | 49 | 13(26.5) | 36(73.5) |  |  |
| CDK4 |  |  |  | 0.081 | 0.776 |
| Negative | 34 | 13(38.2) | 21(61.8) |  |  |
| Positive | 41 | 17(41.5) | 24(58.5) |  |  |
| pPI3K (p85) |  |  |  | 5.471 | 0.019 |
| Negative | 28 | 16(57.1) | 12(42.9) |  |  |
| Positive | 47 | 14(29.8) | 33(70.2) |  |  |
| pAkt-Ser473 |  |  |  | 0.231 | 0.630 |
| Negative | 30 | 13(43.3) | 17(56.7) |  |  |
| Positive | 45 | 17(37.8) | 28(62.2) |  |  |
| pAkt-Thr308 |  |  |  | 8.285 | 0.004 |
| Negative | 34 | 20(58.8) | 14(41.2) |  |  |
| Positive | 41 | 10(24.4) | 31(75.6) |  |  |
| NF-κB (p65) |  |  |  | 0.000 | 1.000 |
| Negative | 20 | 8(40.0) | 12(60.0) |  |  |
| Positive | 55 | 22(40.0) | 33(60.0) |  |  |
| LC3A |  |  |  | 2.250 | 0.134 |
| Negative | 50 | 17(34.0) | 33(66.0) |  |  |
| Positive | 25 | 13(52.0) | 12(48.0) |  |  |
